# Spectral–Spatial Features Integrated Convolution Neural Network for Breast Cancer Classification

**DOI:** 10.3390/s20174747

**Published:** 2020-08-22

**Authors:** Hiren K Mewada, Amit V Patel, Mahmoud Hassaballah, Monagi H. Alkinani, Keyur Mahant

**Affiliations:** 1 Electrical Engineering Department, Prince Mohammad Bin Fahd University, Al Khobar 31952, Saudi Arabia; hmewada@pmu.edu.sa; 2 CHARUSAT Space Research and Technology Center, Charotar University of Science and Technology, Changa, Gujarat 388421, India; amitvpatel.ec@charusat.ac.in (A.V.P.); keyurmahant.ec@charusat.ac.in (K.M.); 3 Department of Computer Science, Faculty of Computers and Information, South Valley University, Qena 83523, Egypt; 4 Department of Computer Science and Artificial Intelligence, College of Computer Science and Engineering, University of Jeddah, Jeddah 21959, Saudi Arabia; malkinani@uj.edu.sa

**Keywords:** biomedical imaging, convolutional neural network, deep learning, wavelet transform, breast cancer classification

## Abstract

Cancer identification and classification from histopathological images of the breast depends greatly on experts, and computer-aided diagnosis can play an important role in disagreement of experts. This automatic process has increased the accuracy of the classification at a reduced cost. The advancement in Convolution Neural Network (CNN) structure has outperformed the traditional approaches in biomedical imaging applications. One of the limiting factors of CNN is it uses spatial image features only for classification. The spectral features from the transform domain have equivalent importance in the complex image classification algorithm. This paper proposes a new CNN structure to classify the histopathological cancer images based on integrating the spectral features obtained using a multi-resolution wavelet transform with the spatial features of CNN. In addition, batch normalization process is used after every layer in the convolution network to improve the poor convergence problem of CNN and the deep layers of CNN are trained with spectral–spatial features. The proposed structure is tested on malignant histology images of the breast for both binary and multi-class classification of tissue using the BreaKHis Dataset and the Breast Cancer Classification Challenge 2015 Datasest. Experimental results show that the combination of spectral–spatial features improves classification accuracy of the CNN network and requires less training parameters in comparison with the well known models (i.e., VGG16 and ALEXNET). The proposed structure achieves an average accuracy of 97.58% and 97.45% with 7.6 million training parameters on both datasets, respectively.

## 1. Introduction

Breast cancer is reported as one of the most leading cause in deaths of women by the International Agency for Research on Cancer (IARC) [1,2,3]. The clinical diagnosis of breast cancer includes inspection of medical images including mammograms, MRI, ultrasound, and histopathology images obtained from a biopsy [4,5]. Among all these, biopsy is the only procedure used for determining a suspicious region by cancer from the breast tissue image. A pathologist analyzes the tissue’s microscopic structure histologically and classifies this structure as normal tissue, benign tissue, and malignant lesions. Variations in normal breast parenchyma’s tissue refer to the benign lesion. The carcinoma tissues can be classified into an in-situ tissue or an invasive tissue. The in-situ tissue contains mammary ductal-lobular inside it, while the invasive carcinoma tissues spread beyond the mammary ductal-lobular structure [6]. The tissue classification is established by examining of the structure of imprints. In some structures, the changes are very elusive causing difficulties in the classification process. Therefore, different magnification factors are utilized by pathologists in analysis and classification of the tissues. This helps to enrich the standard of healthcare with quick and accurate quantification of the tissues. This magnification process requires zooming and focusing on each image and later, scanning of these images entirely for correct diagnosis. This process is a time-consuming and tedious process resulting in delay and sometime inaccuracy in diagnosis.

Classification of signals or images categorize the image into one of the predefined classes [7]. This process is partitioned into two stages: Feature extraction from the images and classification of the features. Features are defined as a characteristic of an image that distinguishes it from the other image class. Previous approaches are based on extraction of traditional features from the images, such histogram of gradient (HoG), local binary pattern (LBP), gray level co-occurrence matrix (GLCM), and SIFT [8]. Then, either supervised or unsupervised algorithms are used to classify these features belongs to any one of the category [9]. Though these approaches have been proven successful in promoting discrimination problems such as healthy and invasive cancer-causing region, the information retrieved by these features is limited for more complex tasks. Supervised classification algorithms like support vector machine and neural network have outperformed the unsupervised classification algorithm including support vector decomposition, principal component analysis, K-mean clustering, and so on.

Since last decade, supervised machine learning algorithms have gained immense popularity and all researchers are extensively using them for computer vision applications [10]. Support vector machine capability to distinguish between the image class highly depends on the feature extraction stage. So, if the extracted features are lacking of representation to distinguish from the classes, then classification accuracy suffers substantially for a given algorithm. A common approach among the past methods following the traditional framework is to pick up multiple features and fuse them intuitively to create more robust features. Yet, it requires both heuristic and manual labor to tweak domain parameters to achieve a decent level of precision. The convolution neural networks (CNNs) integrate these two stages of feature extraction and classification into one black box. The progressive learning from the dataset makes it more robust and achieves better accuracy in contrast to the traditional methods.

The deep architecture of CNN learns hierarchy of features like pixel, edge, pattern, region from the set of training images [11]. CNN based approaches achieved a considerable response in cancer image classification. However, all CNN architectures use predefined convolutional filters which extract the spatial features only. Histopathological image classification depends on the structure/pattern of the cell. The wavelet can discriminate this structure using local features in spatial and frequency domains as well. Therefore, this paper proposes the inclusion of spectral features obtained from the wavelet in CNN for further improvement. The advancement in CNN enabled faster diagnosis of cancer from different magnified histopathological images with higher accuracy. The major confront associated in this classification includes the intrinsic complexity of histopathological images like cell overlapping, irregular color distribution, and subtle variation between images. In this work, a new approach of CNN is developed for cancer image classification between normal, benign, in-situ, and invasive types by concentrating on modifying the CNN architecture considering computation cost. The contributions of this work are summarized as follows:

- A CNN model is proposed utilizing both spectral and spatial information based on a concatenation of multiresolution spectral information obtained from the wavelet transform at the various deep layer of CNN.
- Utilization of average pooling instead of max pooling operation and batch normalization after each convolution operation is introduced to solve the poor convergence problem.
- Performance comparison of the proposed model with various CNN models is presented on two datasets, namely, Breast Cancer Classification Challenge 2015 and BreaKHis.

The rest of this paper is organized as follows. Section 2 provides a brief review of related works on histopathological image classification. Section 3 describes details of fusion spectral and spatial features in CNN for the multi-classification of cancer images. Section 4 presents datasets used in the evaluation and quantitative measures followed by the experimental results. Conclusions are finally given in Section 5.

## 2. Related Work

A binary classification model using a residual learning CNN approach was proposed to learn discriminative features from histopathological images [12]. The algorithm achieved 84.34% and 92.52% classification accuracy without and with augmentation preprocess in the network on the BreaKHis dataset respectively. The Inception recurrent residual convolutional neural network (IRRCNN), a hybrid network consists of residual networks, inception network, and RCNN was created and tested with BreakHis and Breast Cancer Classification Challenge (BCC) 2015 datasets [13] achieving an accuracy of 97.57%. In [14], a dataset from 79 patients was developed and classified using parameter-free Threshold Adjacency Statistics (PFTS) based features and SVM model. Concatenated histogram features are obtained using PFTS and generated a 162-D feature vector. Later, 1-NN and SVM were tested for these datasets. Nahid et al. [15] integrated structural and statistical features using a combination of LSTM and CNN for BCC image classification. In addition to combined NN, they used SVM for binary classification purposes with accuracy limited to 91% for 200× images. Overall the complexity of this structure is very large due to the combination of CNN, LSTM, and SVM.

In the same context, Xie et al. [16] used the transfer learning approach to train the CNN model. They adapted Inception_V3 and Inception_ResNet _V2 for both binary and multi-class classification. The feature size is minimized in Inception_ResNet_V2 using the clustering method, where the K-NN clustering achieves the best results for 2-neighbor and it helps to reduce the feature size. The Inception_ResNet_V2 network outperforms the Inception_V3 network in classification rate. To reduce number of parameters, a small SE-ResNet model was proposed in [17]. Separable filters were utilized in CNN layers providing a light-weight CNN. The algorithm was tested on the BHCNet-3 dataset achieving maximum accuracy of 93.81%. In [18], a prior information from class labels of images have been used to minimize the features distances of cancer images for binary classification and obtained an accuracy of 97% with image augmentation.

A similar approach based on the restricted Boltzmann machine in DNN was introduced in [19]. The contrast of images is enhanced in the pre-processing step using gamma correction and region growing approaches. Texture, tumor shape, and histogram of the image are used as a feature vector in SVM for the classification and the algorithm is validated with a binary classification of BreaKHis images. They summarized that the features belonging to curvature information contribute significantly in the classification in comparison to the other types of features and their achieved accuracy is limited to 89.47%. Mahbod et al. [20] used a transfer learning approach where a natural scene trained two ResNET Neural networks were fine-tuned by modifying the fully connected layer of ResNET. Initially, images are pre-processed, normalized, and then classified using two ResNET networks. A deep CNN with transition layers and dense blocks in contrast to original CNN has been used for BreaKHis and ICIAR image classification and obtained 97.22% maximum accuracy on the BreaKHis dataset. A feature learning based prior information from the structure of images is used in deep CNN by Han et al. [21]. They obtained an average accuracy of 93.2% on the BreaKHis dataset. In [22], ALexNet CNN was adapted and trained from the random patches obtained using a sliding window approach achieved an accuracy of 79.85%. Shen et al. [23] developed a VGG-16 NN with 15 million weight parameters in comparison with ResNET requiring 24 million weight parameters. In this end-to-end training approach, lesion annotations is employed in the early training stage and image-level labels in the later stages. The network is tested on the CBIS-DDSM mammogram dataset and INbreast database achieving 95% AUC.

In [24], the authors used DenseNet where they used concatenation of the features from the previous layer instead of summation. Pre-trained weights obtained from the ImageNet were used in DenseNet and re-trained only a fully connected layer from scratch. The highest achieved accuracy is 96% for multi-classification. Benhammou et al. [25] presented taxonomy on BreakHis dataset by formulating the system using a combination of two classification levels (binary classification and multi-class classification) and dependency on magnification factors (magnification specific and magnification independent). It is reported that histopathological image classification using magnification-independent multi-category is most important than other combination. To avoid class imbalance, data were pre-processed. ImageNet pre-trained ResNet model is used to classify images irrespective of magnification factor and achieved 88.9% accuracy. Kumar et al. [26] used pre-trained VGGNet-16 CNN by removing the fully connected (FC) layer from the network and adding average pooling layer instead of max pooling to extract the features from BreaKHis images. It was reported that polynomial kernels achieved higher accuracy in comparison with linear and RBF kernel. In [27], a fine-grained BreaKHis classification model was proposed using transfer learning approach with Xception model. The architecture was built to multi-task CNN and combined two loss function including Euclidean distance and loss function from the softmax layer to classify images. Sharma and Mehra [28] compared handcrafted features based approach with transfer learning based CNN approach and reported that VGG16 with SVM achieved the best results for the BreaKHis multi-classification task.

In [29], image-wise classification was presented for four classes using CNN. Features were extracted from CNN and classified using a radial basis kernel function based SVM. Experimental results on the BreaKHis dataset showed an accuracy of 90% and 85% for two-class for four-class classification respectively. Zhu et al. [30] assembled multiple CNN networks for the classification. One network obtained features from the patch of images and the second network used downsampled images to obtain features sets. Then, a voting method was used for the classification. Das et al. [31] presented multiple learning CNN framework by aggregating features of the various patches from the same slide in CNN which does not require inter-patch overlap.

## 3. The Proposed Method

The main idea of the proposed method is to fuse the spectral information obtained from the multi-resolution wavelet transform with spatial information obtained using CNN layers. Wavelet transform allows decomposition of an image at various resolution levels providing powerful insight information at frequency level. It helps to scrutinize the local discriminative characteristics in histopathological images [32]. One of the basic wavelet transforms is a Haar wavelet transform. The Haar scaling function and Haar wavelet can be defined by:

(1) $$\phi \left(x\right)=\left\{\begin{array}{cc} 1 & \mathrm{for}\;0\leq x\leq 1\\ 0, & \mathrm{otherwise} \end{array}\right.$$

(2) $$\psi \left(x\right)=\left\{\begin{array}{cc} 1 & \mathrm{for}\;0\leq x\leq 0.5\\ -1 & \mathrm{for}\;0.5\leq x\leq 1\\ 0, & \mathrm{otherwise} \end{array}\right.$$

This can be extended for two-dimensional image analysis, i.e., two-dimensional scaling function and separable decomposition of the wavelets can be expressed as follow

(3) ϕ(x,y)=ϕ(x)ϕ(y)

(4) $$\psi^{H}\left(x,y\right)=\psi \left(x\right)\phi \left(y\right)$$

(5) $$\psi^{V}\left(x,y\right)=\phi \left(x\right)\psi \left(y\right)$$

(6) $$\psi^{D}\left(x,y\right)=\psi \left(x\right)\psi \left(y\right)$$

The discrete wavelet transform of an image I(x,y) with a dimension of (M,N) can be expressed as

(7) $$W_{\phi }\left(j_{0},m,n\right)=\frac{1}{\sqrt{(}MN}\sum_{x=0}^{M-1}\sum_{y=0}^{N-1}I\left(x,y\right)\phi_{j_{0},m,n}\left(x,y\right)$$

(8) $$W_{\psi }^{q}\left(j,m,n\right)=\frac{1}{\sqrt{(}MN}\sum_{x=0}^{M-1}\sum_{y=0}^{N-1}I\left(x,y\right)\psi_{j,m,n}^{q}\left(x,y\right)$$

where, q=H,V,D, $W_{\phi }\left(j_{0},m,n\right)$ is the approximation coefficient of I(x,y) at scale $j_{0}$ and $W_{\psi }^{q}\left(j,m,n\right)$ gives detail coefficients for scales $j>j_{o}$.

**Figure 1:**
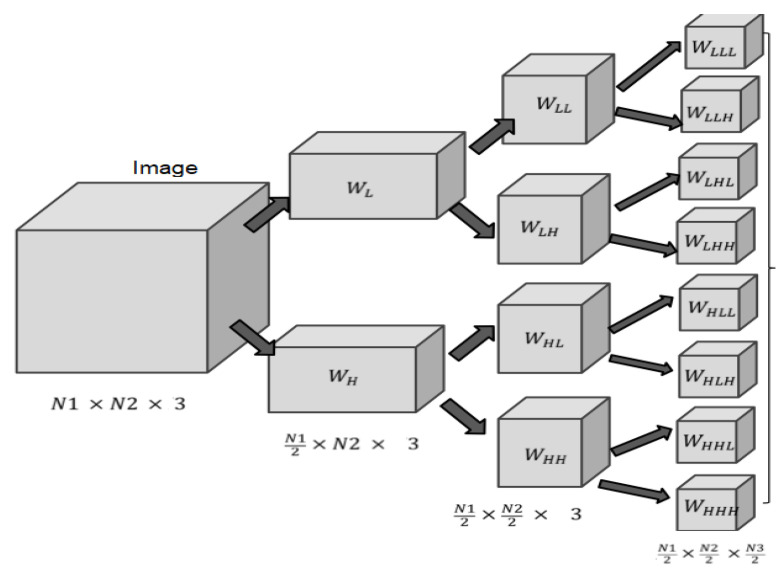
Hierarchical structure of wavelet transform.

Thus, Haar wavelet transform decomposes the image by convolving it with low pass and high pass filter generating coefficients at low-frequency values (approximate coefficients) and high frequency values (horizontal, vertical and diagonal coefficients). The further decomposition of low-frequency values generates next level coefficients at another resolution level. This hierarchical structure of the wavelet transform is shown in Figure 1.

**Figure 2:**
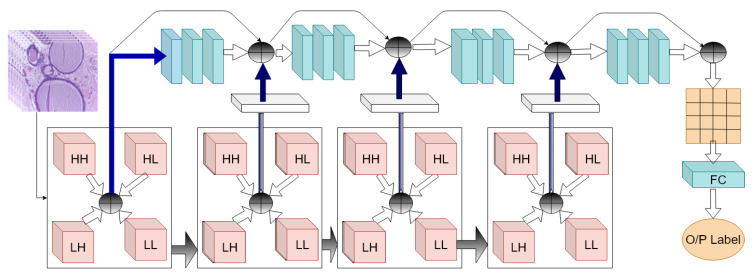
Intuitive block structure of the proposed wavelet-CNN-based method. (⊕ represents concatenation of features)

Kausar et al. [33] preprocessed an image by normalization to remove color variance in images. A 2D-Haar wavelet transform was obtained from these pre-process images. Then, an image obtained from the second level decomposition was used in VGG-16 CNN network for classification. Thus, they are not utilizing all multi-resolution features obtained from the DWT. In contrast, the proposed method fuses features obtained from all resolution in Deep CNN. An intuitive block diagram of the proposed method is shown in Figure 2. On the other hand, the success of a convolutional neural network (CNN) depends on the number of parameters and hidden layers and the number of images available for training. The VGG-16 requires 138 million parameters. ResNet [34] and DenseNet [35] models achieve considerably better performance on large size ImageNet (10 million images, 1000 categories) dataset [36], they need more memory and computations compared to VGG16-net. In contrast to these models, the proposed model has a total 7.6 million parameters including 13,440 non-trainable parameters. The detailed structure is presented in Table 1.

Performance of wavelet level decomposition at level 6 is better than others irrespective of decomposition type [37]. After decomposition level 6, the modeling accuracy becomes stable (i.e., marginal improvement). In the proposed structure, the wavelet transform is obtained for four decomposition levels over histological image size of 512,512×3. Various numbers of filters with size of 3×3 are used in each convolutional layer of the model, i.e., 64 in layer 1, 64 in layer 2, 128 in layer 3, and so on. The batch normalization process is used after every layer in the convolution network to improve the poor convergence problem of CNN. Additionally, to increase the speed of the training process, an activation function Rectified Linear unit (ReLU) is utilized after normalization. Further, the max-pooling operation is used to reduce the future vector size from the output of the activation function in the CNN network. The average pooling operation can be expressed as

(9) Y=(I∗P)↓p

where, *I* is the input image, *P* is the average filter and p=2 is stride. In the proposed model, the wavelet transform fulfills the requirement of the pooling operation. The Haar wavelet transform is obtained by convolving the image with a low pass filter $W_{LL}$ to obtain low-frequency coefficients and three high pass filter $W_{LH},W_{HL},W_{HH}$ giving high-frequency coefficients. For Haar wavelet, these filters are defined as

(10) $$W_{LL}=\left[\begin{array}{cc} 1 & 1\\ 1 & 1 \end{array}\right],W_{LH}=\left[\begin{array}{cc} -1 & -1\\ 1 & 1 \end{array}\right],W_{HL}=\left[\begin{array}{cc} -1 & 1\\ -1 & 1 \end{array}\right],W_{HH}=\left[\begin{array}{cc} 1 & -1\\ -1 & 1 \end{array}\right]$$

Therefore, wavelet transform can be represented equivalent to pooling operation as

(11) $$X_{ij}=I*W_{ij}|2$$

Instead of using a fixed average filter in the average pooling operation, the wavelet transform uses four filters with stride 2. This down-samples the size of the features by 2. To get the advantage of both spectral as well as spatial information the concatenation of wavelet features and spatial features obtained from the convolution layer is carried out.

## 4. Experiment Results

### 4.1. Datasets

**Figure 3:**
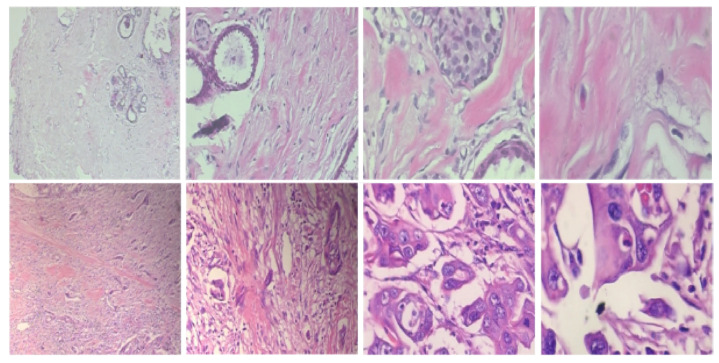
Sample images of the BreaKHis dataset for benign (first row) and malignant (second row) with zooming of 40×, 100×, 200×, and 400× (left to right, respectively).

**Figure 4:**
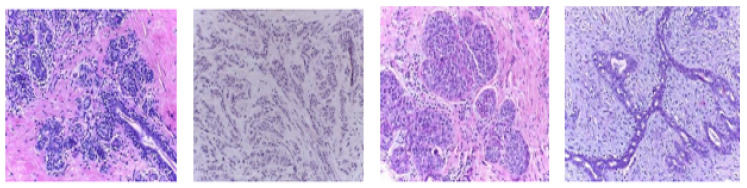
Sample images of the BCC2015 dataset, left to right: Normal, benign, in situ carcinoma, and invasive carcinoma.

In this work, the histopathological images are augmented and then the model is trained using this augmented dataset. The performance of the model is evaluated on two publically available datasets, namely BreaKHis dataset [14] and Breast Cancer Classification Challenge 2015 (BCC2015) [38]. The BreaKHis dataset contains a total of 7909 images including 2480 benign images and 5429 malignant images with four magnification factors of 40×, 100×, 200×, and 400×. All images have an RGB color map with a 700×460 resolution. Sample images of the BreaKHis dataset are shown in Figure 3. The BCC2015 dataset has a total of 5229 images including 1155 normal images, 1449 benign images, 1323 In situ, and 1302 invasive images with 2040 × 1536 resolution. Sample images of the BCC2015 dataset are shown in Figure 4. Experiments are conducted using patch-wise evaluation. It should be noted CNN cannot be used with images of high resolution (i.e., entire slide tissue images). Moreover, applying CNN to such high resolution images requires downsampling process. However, it loses the most discriminative information. To encode these discriminative information, images are partitioned to patches of size 512 × 512.

### 4.2. Data Augmentation

**Figure 5:**
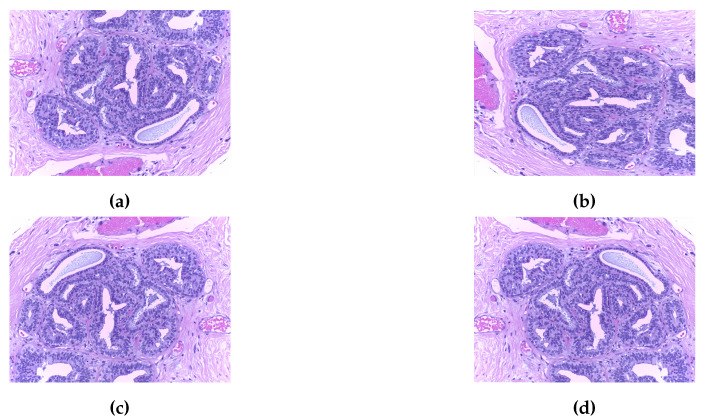
Patches augmentation: (**a**) Original image. (**b**) Image at 90° rotation. (**c**) Image at 180° rotation. (**d**) Flipped image using vertical mirroring.

The network is likely to overfit with a small dataset. Therefore, training images have been increased using data augmentation, where, the images have been divided into number of patches and rotation. Then, mirroring and shifting operations on patches are used to augment dataset. Image patching and augmentation have been used well on histological images classification [39]. Rotation and shifting operation allows classification of images at various orientation while mirroring operation allows increasing the number of samples without deteriorating its features. The patches of 512×512 pixels are obtained from the images with a 50% overlap. Some example of augmented patches are shown in Figure 5. Each patch is normalized by subtracting the mean value to the color channels separately. Then, the patch is altered into eight patches using the rotation of 0,π/2,π,3π/2, and vertical mirroring. The label associated with the patches is the same as the original image.

### 4.3. Evaluation Metrics

To quantify and validate the performance of the proposed method, well-known metrics, namely, classification accuracy, area under the curve, sensitivity and specificity are used. For classification problem, a predicted output can be classified into four states. (a) True Positive (TP) suggest that image is classified as benign correctly, i.e., both label and classification are benign type (b) False Positive (FP) suggest that image is wrongly classified as benign type. That is the label is not benign and classification is benign type (c) True Negative (TN) suggest that both label and classification are not benign (d) False negative (FN) suggest wrong classification, which means image label is benign and classified as malignant. Using these parameters, sensitivity (also referred as True Positive Rate (TPR)) is defined as ability of the algorithm to correctly identify images with diseases and Specificity defines the ability of the algorithm to correctly classify image without diseases. Mathematical formulation of these metrics are as follows:

(12) $$Accuracy=\frac{\mathrm{Number}\;\mathrm{of}\;\mathrm{correct}\;\mathrm{classification}}{\mathrm{Total}\;\mathrm{Number}\;\mathrm{of}\;\mathrm{classification}}=\frac{TP+TN}{TP+TN+FP+FN}$$

(13) $$Sensitivity=\frac{\mathrm{No}\;\mathrm{of}\;\mathrm{true}\;\mathrm{positive}\;\mathrm{classification}}{\mathrm{Total}\;\mathrm{No}\;\mathrm{of}\;\mathrm{all}\;\mathrm{positive}\;\mathrm{classification}}=\frac{TP}{TP+FN}$$

(14) $$Specificity=\frac{\mathrm{No}\;\mathrm{of}\;\mathrm{true}\;\mathrm{negative}\;\mathrm{classification}}{\mathrm{Total}\;\mathrm{No}\;\mathrm{of}\;\mathrm{all}\;\mathrm{negative}\;\mathrm{classification}}=\frac{TN}{TN+FP}$$

The tradeoff between the specificity and sensitivity can be evaluated using receiver operating characteristic (ROC). Thus increment in the sensitivity values causes decrements in the specificity. The the area under the ROC (AUC) depicts the balance between these two attributes. The large AUC indicates the better separability between the classes by the algorithm. The following subsections represent the performance analysis of proposed algorithm for both binary classification and multi-class classification using these attributes.

### 4.4. Performance Analysis on the Breakhis Dataset

**Figure 6:**
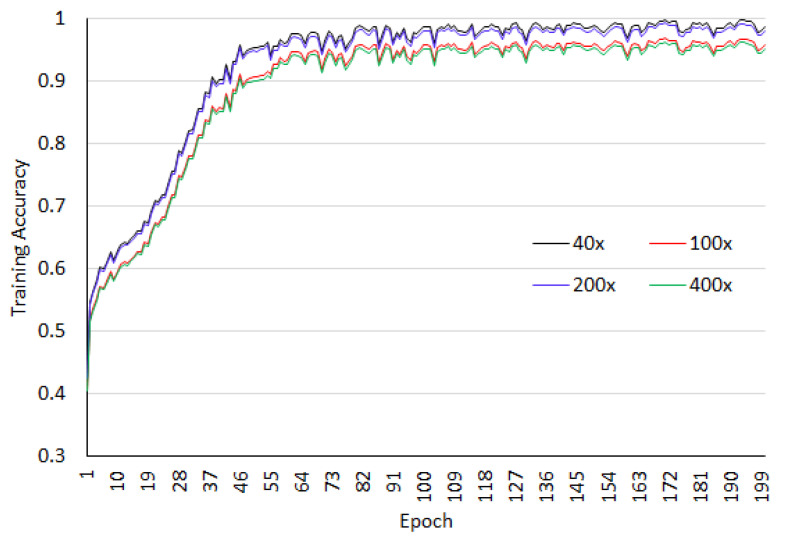
Training accuracy for the breaKHis dataset.

**Figure 7:**
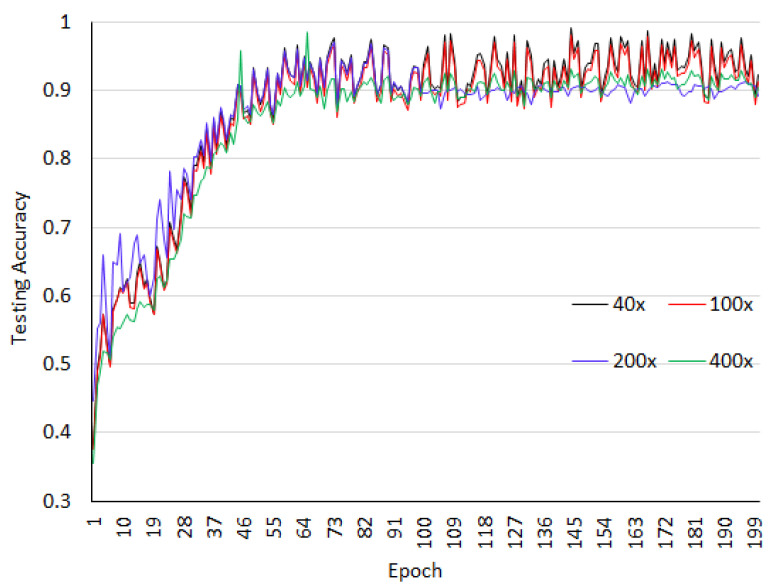
Testing accuracy for the breaKHis dataset.

The dataset is arbitrarily divided into 70% training dataset and 30% testing dataset. All images patches are resized to 512×512. Four level wavelet decomposition is used in the experiment. The network is trained for 200 epochs and 3 batch sizes. As listed in the Table 1, the configured network requires 76,289,732 trainable parameters and 13,440 non-trainable parameters. For the BreaKHis dataset, binary classification is analyzed. The accuracy analysis for the training as well as test datasets at different magnification factors of images for BreaKHis are shown in Figure 6 and Figure 7. We observed from these figures that the magnification has an impact on the classification accuracy and for 40× and 100× better accuracy is obtained.

**Figure 8:**
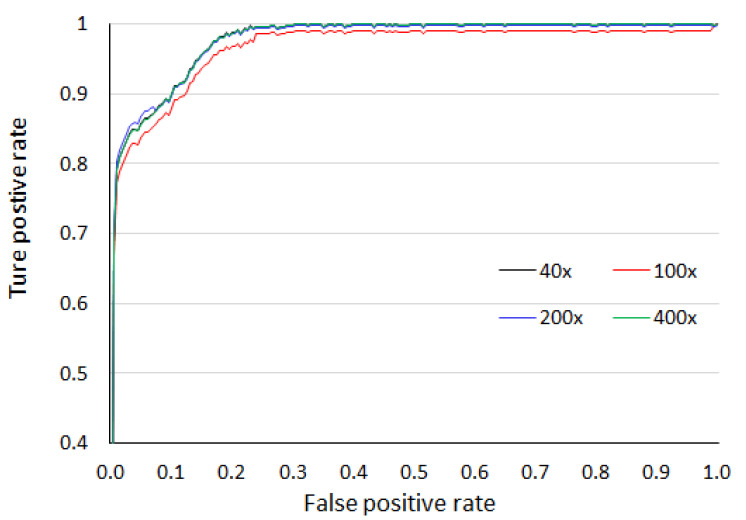
Area under the curve over four magnification factors for the breaKHis dataset.

Figure 8 gives the region of convergence graph with consideration of the area under the curve. The obtained AUC values are 99.49%, 99.20%, 99.33% and 99.40% for 40×, 100×, 200×, and 400× magnification factors, respectively. The comparative analysis of the obtained accuracy with state-of-art methods is presented in the Table 2. For 40× magnification, the proposed method achieves the highest accuracy among all whereas, for remaining magnification, the accuracy is better or comparable with other methods.

### 4.5. Performance Analysis on the Bcc2015 Dataset

**Figure 9:**
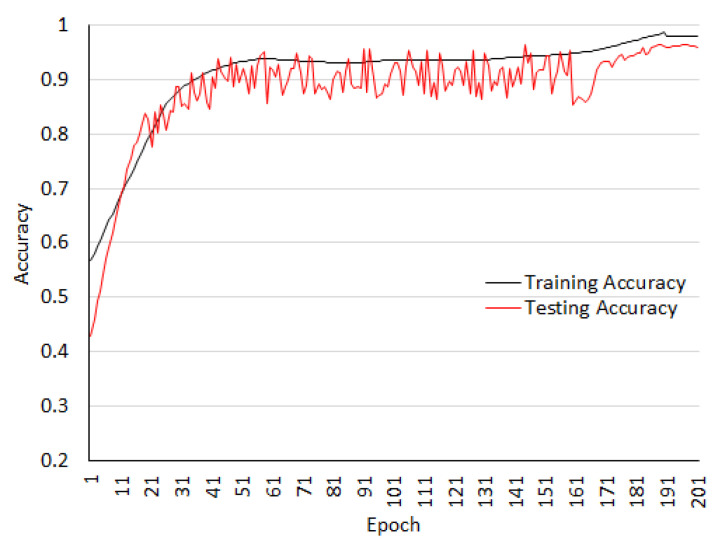
Training and testing accuracy on the BCC2015 dataset.

The experimental results for the BCC2015 dataset are conducted for multi-class classification, where images are classified between the four class (normal, benign, in-situ, and invasive type). The same model with same parameters has been used in the experiment. Images are augmented as previous described. The training and testing accuracy for this dataset are shown in the Figure 9. The graph shows that the training accuracy and testing accuracy are matching.

Comparison with state-of-art methods is also reported in Table 3. The proposed model achieves comparable results with IRRCNN model [13]. The hybrid CNN architecture has strong classification power but requires large memory and more computing resources which prompts higher diagnosing dormancy in some genuine clinical applications. In [16], the last fully connected layer of Inception_ResNet_V2(IRV2) architecture trained using ImageNet dataset for histopathology image classification is modified to reduced feature dimension by passing the features obtained from the IRV2 to the autoencoder network. However, the IRV2 architecture requires 572 depth with 55 million of learnable parameters. It should be noted that IRRCNN is a hybrid CNN architecture consisting of inception network, recurrent CNN and residual network. The inception network concatenates the features obtained from convolutional operation with different size of the kernels. Then features obtained from this inception unit are added to the input features of respective unit forming inception- residual network. Furthermore, They the recurrent structure is formed, where features obtained at the current time stamp are added with the features of the past time stamp. Thus, the IRRCNN model has large computational complexity in comparison with the proposed wavelet features concatenated CNN architectures and it has 9.3 million learnable parameters. The IRRCNN architecture was implemented with 56G of RAM and an NVIDIA GEFORCE GTX-980 Ti processor. In contrast, the proposed architecture is implemented on the i7 processor with 8GB RAM and it has 7.6 million learnable parameters. Therefore, that the proposed architecture deviates with a fraction of percentage in recognition accuracy with 1.2 times less learnable parameters in comparison with IRRCNN.

## 5. Conclusions

In this paper, we proposed a method for histopathological cancer image classification based on a modified CNN model. The weakness of the traditional CNN model is that its classification depends on the spatial features only that can be obtained from the training dataset. However, the spectral features play an equivalent role to the spatial features in the classification. Hence, the CNN model is modified and Haar wavelet-based spectral features are fused with spatial features to enhance the performance of the classifiers. Two databases, breaKHis dataset and BCC2015, are used in the experiments with different criteria of magnification factor, augmented patches, binary classification, and multi-class classification. The proposed model achieved an average accuracy of 97.58% and 97.45% on the breaKHis dataset and BCC2015 dataset, respectively, which is higher than most of the state-of-art methods. It is also observed that it requires only 7.6 million learning parameters, which proposes a design of a lightweight CNN algorithm with inclusion of spatial and spectral information. Future research will focus on testing other wavelet families, such as Daubechies, Biorthogonal, Coiflet, which may have good capability in structure discrimination.

## Figures and Tables

**Table 1:** Details of the proposed Convolution Neural Network (CNN) model layer connection with wavelet transform.

Layer (Type)	Output Shape	Parameters	Connected to
InputLayer	(512,512,3)	0	
wavelet	(256,256,12)	0	InputLayer
conv_1	(256,256,64)	6976	wavelet
norm_1	(256,256,64)	256	conv_1
relu_1	(256,256,64)	0	norm_1
conv_1_2	(128,128,64)	36,928	relu_1
conv_a	(128,128,128)	13,952	wavelet[1]
norm_1_2	(128,128,64)	256	conv_1_2
norm_a	(128,128,128)	512	conv_a
relu_1_2	(128,128,64)	0	norm_1_2
relu_a	(128,128,128)	0	norm_a
concate_1	(128,128,192)	0	relu_1_2, relu_a
conv_2	(128,128,128)	221,312	concate_1
conv_b	(64,64,64)	6976	wavelet[2]
norm_2	(128,128,128)	512	conv_2
norm_b	(64,64,64)	256	conv_b
relu_2	(128,128,128)	0	norm_2
relu_b	(64,64,64)	0	norm_b
conv_2_2	(64,64,128)	147,584	relu_2
conv_b_2	(64,64,128)	73,856	relu_b
norm_2_2	(64,64,128)	512	conv_2_2
norm_b_2	(64,64,128)	512	conv_b_2
conv_c	(32,32,256)	27,904	wavelet[3]
relu_2_2	(64,64,128)	0	norm_2_2
relu_b_2	(64,64,128)	0	norm_b_2
norm_c	(32,32,256)	1024	conv_c
concate_2	(64,64,256)	0	relu_2_2 relu_b_2
relu_c	(32,32,256)	0	norm_c
conv_3	(64,64,256)	590,080	concate_2
conv_c_2	(32,32,256)	590,080	relu_c
nomr_3	(64,64,256)	1024	conv_3
norm_c_2	(32,32,256)	1024	conv_c_2
relu_3	(64,64,256)	0	nomr_3
relu_c_2	(32,32,256)	0	norm_c_2
conv_3_2	(32,32,256)	590,080	relu_3
conv_c_3	(32,32,256)	590,080	relu_c_2
norm_3_2	(32,32,256)	1024	conv_3_2
norm_c_3	(32,32,256)	1024	conv_c_3
relu_3_2	(32,32,256)	0	norm_3_2
elu_c_3	(32,32,256)	0	norm_c_3
concate_3	(32,32,512)	0	relu_3_2 relu_c_3
conv_4	(32,32,256)	1179904	concate_3
relu_4	(32,32,256)	0	norm_4
conv_4_2	(16,16,256)	590,080	relu_4
norm_4_2	(16,16,256)	1024	conv_4_2
relu_4_2	(16,16,256)	0	norm_4_2
conv_5_1	(16,16,128)	295,040	relu_4_2
norm_5_1	(16,16,128)	512	conv_5_1
relu_5_1	(16,16,128)	0	norm_5_1
pool_5_1	(16,16,128)	0	relu_5_1
flat_5_1	(32768)	0	pool_5_1
fc_5(Dense)	(2048)	67,110,912	flat_5_1
norm_5	(2048)	8192	fc_5
relu_5	(2048)	0	norm_5
drop_5	(2048)	0	relu_5
fc_6	(2048)	4,196,352	drop_5
norm_6	(2048)	8192	fc_6
relu_6	(2048)	0	norm_6
drop_6	(2048)	0	relu_6
fc_7	(4)	8196	drop_6

**Table 2:** Comparative analysis of binary classification accuracy (%) with other methods on the BreaKHis dataset.

Methods	40×	100×	200×	400×
ResHist model [12]	86.38	87.28	91.35	86.29
**IRRCNN w/o augmentation [13]**	**97.16**	**96.84**	**96.61**	**95.78**
**IRRCNN with augmentation [13]**	**97.95**	**97.57**	**97.32**	**97.36**
Alex Net [22]	85.6	83.5	82.7	80.7
class structure-based deep CNN [21]	92.8	93.9	93.4	92.9
Multi task CNN [40]	81.87	83.39	82.56	80.69
CNN & Fusion Rules [41]	90.0	88.4	84.6	86.1
VLAD encoding [42]	91.8	92.2	91.6	90.5
Structured Deep Learning [43]	95.8	96.9	96.7	94.9
**IRV2+1-NN_Aug [16]**	**98.04**	**97.50**	**97.85**	**97.48**
RBM [15]	88.7	85.3	88.6	88.4
DenseNet CNN [24]	93.64	97.42	95.87	94.67
PFTS Features + 1-NN [14]	80.9	80.7	81.5	79.4
PFTS Features + SVM [14]	81.6	79.9	85.1	82.3
VGGNET16-RF [26]	92.22	93.40	95.23	92.80
VGGNET16-SVM(POLY) [26]	94.11	95.12	97.01	93.40
Xception model [27]	95.26	93.37	93.09	91.65
**Proposed method**	**97.58**	**97.44**	**97.28**	**97.02**

**Table 3:** Comparison of multi-class classification accuracy (%) on the BCC2015 dataset.

Model	Sensitivity	Specificity	Accuracy	AUC
CNN [29]	61.1	94.4	77.8	-
CNN+ SVM [29]	66.7	65.6	83.3	-
IRRCNN with augmentation [13]	97.71	98.89	98.59	99.05
multiple compact CNNs [30]	-	-	86.6	93.91
Proposed Wavelet + CNN	96.59	97.73	97.45	99.03
